# Autophagy/ Apoptosis Induced by Geraniol through HIF-1α/BNIP3/Beclin-1 Signaling Pathway in A549 CoCl2 Treated Cells

**DOI:** 10.34172/apb.2022.016

**Published:** 2020-10-18

**Authors:** Dina M. Abo El-Ella

**Affiliations:** Department of Pharmacology and Toxicology, Faculty of Pharmacy, October 6 University, 6 October City, 12566, Giza, Egypt.

**Keywords:** Hypoxia, Geraniol, HIF-1α, Autophagy, BNIP3, Beclin-1, VEGF

## Abstract

***Purpose:*** During cancer growth, hypoxia occurs along with autophagy as an adaptive responseto overcome cellular stress. Geraniol (GE) is a natural isoprenoid known for its wide anticanceractivity and autophagy induction in the cancer cell. To investigate the antihypoxic potential ofGE with the incidence of autophagy and apoptotic cell death in A549 CoCl_2_ treated cells.

***Methods:*** A549 cells were incubated for 24 hours with GE and CoCl_2_ either alone or incombination. We examined the cytotoxicity and cell viability of GE either alone or incombination therapy using MTT and trypan blue assay.GE modulating effect was determined onlipid peroxidation, antioxidant capacity markers, gene expression levels of hypoxia induciblefactor-1 (HIF-1), NF-κB, vascular endothelial growth factor (VEGF), autophagy factors in differentgroups, besides apoptotic bodies using acridine orange/ethidium bromide (AO/EB).

***Results:*** GE and CoCl_2_ combination therapy downregulated the expression of HIF-1α thatsuppressed A549 cell growth through downregulation of BNIP3 and beclin-1 gene expression.This resulted in autophagy and apoptotic cell death, in addition to the downregulation of NF-kBand VEGF expression. Also, GE treatment significantly reduced the oxidative stress markers andrestored the antioxidant capacity.

***Conclusion:*** GE possesses an antihypoxic effect on A549 CoCl_2_ treated cells and induces celldeath via autophagy along with apoptosis through HIF-1α/BNIP3/beclin-1 signaling pathway.

## Introduction

Lung cancer is one of the leading causes of death worldwide. This is due to the formation of wide areas of hypoxic tissue that reduce the response to radiotherapy and chemotherapy.^1^ Therefore, developing anti-hypoxic drugs is the main aim of lung cancer therapy by targeting hypoxia, concurrent sequences of angiogenesis, invasiveness, and tumor growth.^2^

Hypoxia and necrotic areas are developed from insufficient oxygen supply during solid tumor growth.^3^ Hypoxia inducible factor-1 (HIF-1) is the main cellular regulator during hypoxia, composed of oxygen regulated HIF-1 α subunit as well as the constitutively expressed HIF-1 β subunit.^4,5^ HIF-1 α role during hypoxia is to maintain blood, nutrients as well as energy resumption through the expression of regulatory genes controlling glycolysis, angiogenesis, and erythropoiesis.^6,7^ Recent studies reported a bi-directional relation between HIF-1 α and NF-κB because HIF regulates the expression of NF-κB mRNA and vice versa.^8^

Autophagy is a self-eating and dynamic phenomenon that depends on lysosomal degradation of the damaged mitochondria, cytoplasm, lipids, and misfolded proteins back to their basic components with cellular recycling to preserve cellular homeostasis.^9,10^ Autophagy is present at a low level under physiological conditions but increased under cellular stress ailments including hypoxia, and nutrient deficiency and ROS production.^10-12^ Autophagy and apoptosis share many regulators and interactions like beclin-1/Bcl-2,^13^ and caspase/beclin-1 cleavage.^14^ During moderate hypoxic conditions, HIF-1α induces activation of autophagy in a dependent manner and supplies cancer cells with cellular energy requirement for solid tumor proliferation.^15,16^ However, severe hypoxia induces autophagy in HIF-1α independent manner through involvement of the AMPK-mTOR and unfolded protein response pathways.^15,17^ BNIP3 and beclin-1 (Bcl-2 gene family) are cell death proteins that play an important role in hypoxia associated apoptosis, necrosis, and autophagy.^18-20^ Thus, BNIP3 and beclin-1 are markers for appraising autophagy.

Geraniol (GE) is a natural isoprenoid monoterpene derived from the essential oils of various aromatic plants including lemon, ginger, rose, and orange.^21^ Previous *in vitro* and *in vivo* studies showed that GE possesses anti-microbial, anti-inflammatory, and anti-oxidantproperties.^22,23^ Moreover, GE exerts an apoptotic, antitumor, and anti-angiogenic effect on various cancer cells,^24-26^ besides being a potent inducer of autophagy.^27^

Taking these previous pharmacological effects into account, this study was designed to determine the protective effect of GE against CoCl_2_ induced hypoxia associated autophagy on the A549 cell line and focused on the relation between HIF-1 α, autophagy, and apoptotic cell death.

## Materials and Methods

### Media and reagents

All chemicals and reagents were obtained from Sigma Aldrich Chemical Co. (St. Louis, MO, USA) unless otherwise stated during this study. This study was achieved at the Cell Culture Research Unit on October 6 University (Cairo, Egypt).

### Cell line and cell culture

Human lung adenocarcinoma epithelial A549 cells (ATCC, USA) were cultured according to the American Type Culture Collection protocol.^28^ All experiments were repeated three times independently, and the data were represented as the mean ± standard deviation (SD).

### MTT assay

A549 cells were grown to 90% confluence then undergo trypsinization followed by counting with a hemocytometer. Afterward, A549 cells were seeded in 96 well tissue culture plates at 10 × 10^3^/well in triplicates for 24 hours for cell attachment before further additions. The A549 cells were treated with various concentrations of GE (0.161 to 2.59mM) and CoCl_2_ (0.193 to 7.75mM) either alone or in combination then incubated for 24 hours.^29,30^ After treatment, the standard 3,4,5-dimethylthiazol-2,5- diphenyl tetrazolium bromide (MTT) method was used to assess the cytotoxicity of GE alone or its combination with CoCl_2_.^31^ The IC_50_ concentration of GE and CoCl_2_ either alone or in combination on the A549 cell line after 24 hours were determined and used to assess other bioassays. The mean percentages of cell viability were detailed as mean ± SD.

### Cell viability by the trypan blue dye exclusion assay

Cell viability of A549 cells in treated and untreated groups was determined by the trypan blue assay that depends on quantify live cells by labeling dead cells exclusively. The A549 cells were stained with 10 μL trypan blue dye (0.4% solution) and to count unstained live and blue stained dead cells under a phase contrast microscope with a hemocytometer.^32^ The mean percentages of live and dead cells per experiment were expressed as mean ± SD of three autonomous experiments.

### Apoptosis and necrosis staining

Acridine orange/ethidium bromide staining (AO/EB) used to detect the mode of cell death.^33^ The mean percentages of live, apoptotic, and necrotic cells per experiment were determined according to Zakaria and colleagues and were expressed as mean ± SD.^34^

### Cell lysate preparation

The preparation of cell lysate was done according to Zakaria and colleagues to be used in the analysis of oxidative stress and inflammatory markers.^34^ Bicinchoninic acid used to determine the protein content of total cell lysate using bovine serum albumin as a standard, data not cited.^35^

### Estimation of oxidative stress markers (MDA and TAC)

Lipid peroxidation of cell lysate of treated and untreated groups was determined using malondialdehyde (MDA) as a representative of its final product using a commercially supplied kit (Biodiagnostic).^36^ Also, the total antioxidant capacity (TAC) of the cell lysate of different groups was assayed colorimetrically according to the protocol of the Biodiagnostic supplied kit.^37^

**Gene** ***expression analysis using quantitative real-time polymerase chain reaction (*** ***qRT*** ***-PCR)***

qRT-PCR was used to quantify the changes in HIF-1α, NF-κB, vascular endothelial growth factor (VEGF), BNIP3, and beclin-1 mRNA expression in different groups. In ΔΔCT method, GAPDH mRNA was used as the internal control to detect the variation in the genes expression of HIF-1α, NF-κB, VEGF, BNIP3, and beclin-1 in all groups.^38^ All primer sequences used in amplifying the HIF-1α, NF-κB, VEGF, BNIP3, and beclin-1 genes are listed in Table 1.

**Table 1:** Primer sequences used in qRT-PCR analysis

**Target gene**	**Primer sequences**
GAPDH	F: CTCTGATTTGGTCGTATTGGG
	R: TGGAAGATGGTGATGGGATT
NF-κB	F: TGGTGCCTCACTGCTAACT
	R: GGATGCACTTCAGCTTCTGT
HIF-1α	F: ATCCATGTGACCATGAGGAAATG
	R: TCGGCTAGTTAGGGTACACTTC
VEGF	F: AGGGCAGAATCATCACGAAGT
	R: AGGGTCTCGATTGGATGGCA
BNIP3	F: CAGGGCTCCTGGGTAGAACT
	R: CTACTCCGTCCAGACTCATGC
Beclin-1	F: GGCTGAGAGACTGGATCAGG
	R: CTGCGTCTGGGCATAACG

### Statistical analysis

Experimental data were analyzed by GraphPad Prism (ISI®, USA) software (version 5) and expressed as mean ± SD. One-way ANOVA followed by Tukey Kramer as a post hoc test was used to detect the variation between groups. A *P* value of less than 0.05 was considered a statistically significant difference. (*) is significant compared with the A549 cells, and (#) is significant compared with the CoCl_2_ treated group.

## Results

### Cytotoxicity of GE and CoCl_2_ either alone or in combination

**Figure 1:**
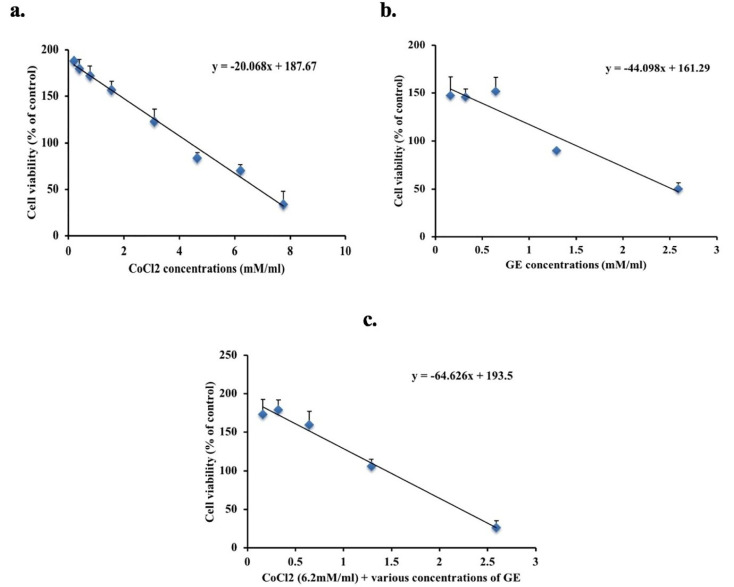
MTT assay used to determine the cytotoxic effect of various concentrations of GE (b) and CoCl2 (a) either alone or in combination (c) on A549 cells for 24 h. Data expressed as a percentage of untreated cells. The figure shows averages from triplicate data of three separate experiments and expressed as mean ± SD.

A significant decrease in cell viability in the CoCl_2_ (hypoxic) and GE groups was observed compared to untreated cells and the calculated IC_50_ was 6.2mM and 2.59mM subsequently, as shown in Figure 1a-b. The effect of combined therapy was assessed on the viability of A549 using the IC_50_ of CoCl_2_ added to various concentrations of GE for 24 hours. As shown in Figure 1c, the combination therapy of CoCl_2 _+ 2.59mM GE showed a significant reduction in cell viability (26 % ± 9) compared to untreated cells. Contrary to this, CoCl_2 _+ 1.29mM of GE showed the least reduction in cell viability. Thus, the IC_50_ value of GE (2.59mM) and its diluted concentration ½ IC_50_ 1.29mM will be used in the combination therapy with IC_50_ of CoCl_2_ for 24 hours in the following bioassays.

### Trypan blue dye exclusion assay

**Figure 2:**
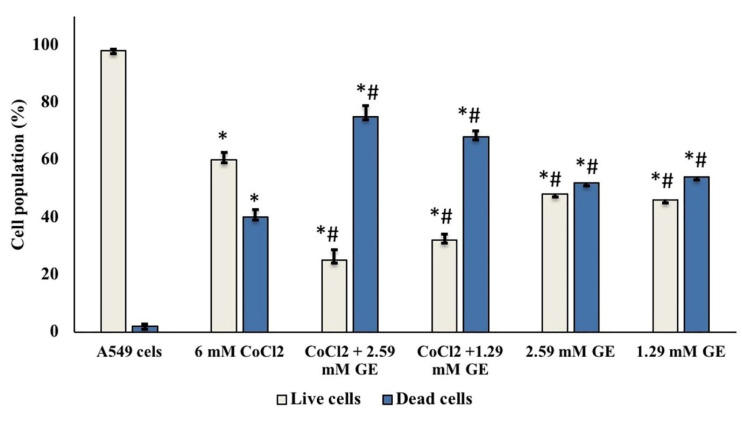
The cell viability assessment of A549 cells treated with GE and CoCl_2_ either alone or in combination after 24 h incubation, stained with trypan blue dye under a phase-contrast microscope; data showed mean (%) ± SD; n = 3; (light bars) demonstrates cell population percentages of viable cells; (dark bars) demonstrated cell population percentages of dead cells. (*) significantly different from A549 group (*P* < 0.05), (#) significantly different from 6.2mM CoCl_2_ group (*P* < 0.05).

As shown in Figure 2, A549 cells treated with GE (2.59 and 1.29mM) showed a significant decrease in the number of viable cells 48% ± 2.6, and 46% ± 3.2 respectively regarding that noticed in untreated cells (98% ± 0.6). Also, CoCl_2_ A549 treated cells (6.2mM) either alone or in combination, showed a major reduction in the number of viable cells (60% ± 2.6, 25% ± 3.8, and 32% ± 2.1 respectively) compared to that observed in untreated cells.

### Apoptosis and necrosis staining

**Figure 3:**
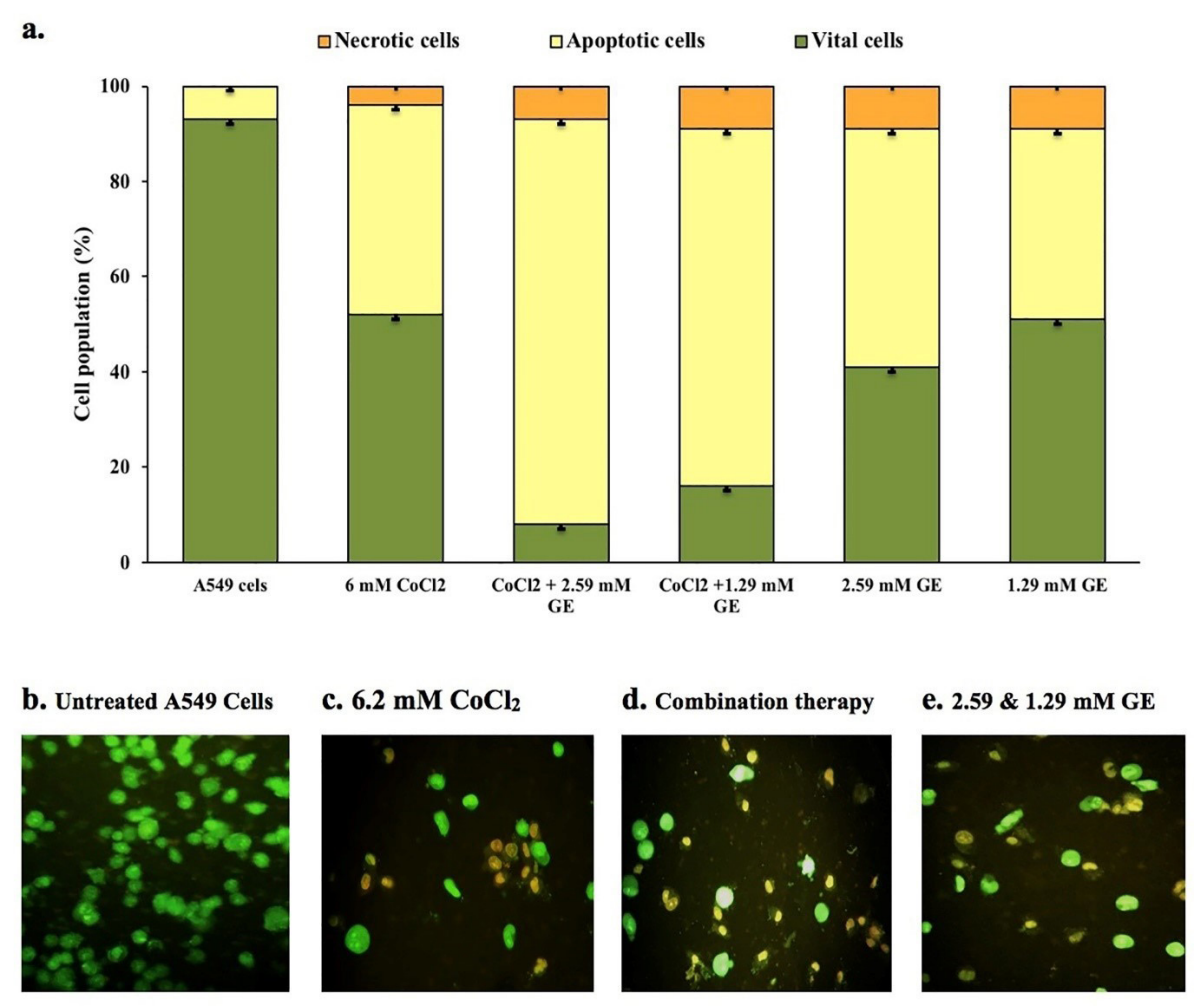
Fluorescence microscopic images (x200) of apoptotic and necrotic cell death induced by GE 2.59 and 1.29mM) and CoCl_2_ (6.2mM) either alone or in combination in A549 cells. Data showed mean (%) ± SD; n = 3; (a) demonstrates cell population percentages of apoptotic-necrotic cells; (b) vital cells (green), (c,d) apoptotic cells (yellow), (e) early apoptotic bodies (bright yellowish-green).

Under a fluorescence microscope, dual DNA staining by AO/EB dyes was used to determine the mode of cell death in untreated and treated A549 cell line. The untreated group showed no sign of cell death and most of the cells are viable with uniform green fluorescence color (Figure 3a-b). CoCl_2_ treated group showed a high number of yellow to orange stained cells with a mean percentage of apoptotic cell death of 44% ± 1.5 compared to that noticed in untreated cells (93% ± 1) (Figure 3a-c). Groups treated with GE (2.59 and 1.29mM) and CoCl_2_ showed a significant percentage of apoptotic cell death (85% ± 2 and 75% ± 2.5 respectively) compared to that detected in CoCl_2_ treated group as well as few necrotic cells that display orange to red nuclei (Figure 3d). Moreover, GE treated groups showed a meaningful percentage of apoptotic cells (50% ± 1 and 40% ± 1.5 respectively) with bright green to yellow color compared to untreated groups (Figure 3e).

### Oxidative stress marker and antioxidant activity

**Figure 4:**
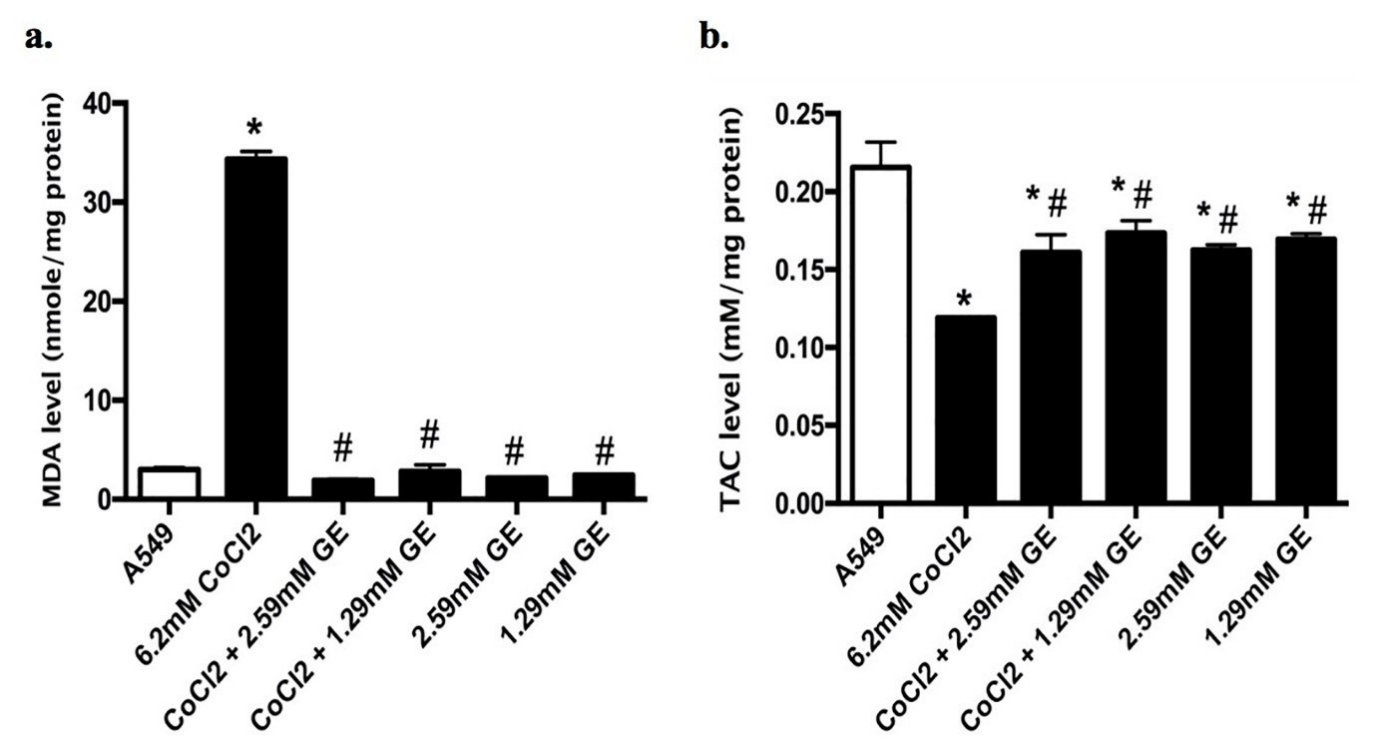
Levels of (a) MDA and (b) TAC in A549 cells treated with GE (2.59 and 1.29mM) and CoCl2 6.2mM) either alone or in combination for 24 h exposure using ELISA Kit. Data expressed as nM/mg protein. (*) significantly different from A549 group (*P* < 0.05), (#) significantly different from 6.2mM CoCl2 group (*P* < 0.05). The figure shows the mean ± SD of averages from triplicate data of three separate experiments.

Combined therapy of GE (2.59 and 1.29mM) and CoCl_2_ (6.2mM) on A549 for 24 hours showed a significant reduction in lipid peroxidation in terms of MDA level (1.9 ± 0.1 and 2.8 ± 0.6 respectively) compared to that observed in CoCl_2_ treated cells (34.4 ± 0.75); MDA is an eminent detector for oxidative stress (Figure 4a). On the other hand, TAC in untreated and treated groups was determined to detect the effect of ROS generation on antioxidant system. Combination therapy of GE (2.59 and 1.29mM) and CoCl_2_ as well as GE treated groups showed a significant elevation in the TAC levels (0.161 ± 0.01, 0.173 ± 0.007, 0.16 ± 0.003, and 0.169 ± 0.003 respectively) compared to that noticed in CoCl_2_ treated cells (0.11 ± 0.01) as presented in Figure 4b.

### Relative expression of HIF-1α, NF-κB, VEGF, BNIP3, and Beclin-1 mRNA in different groups

**Figure 5:**
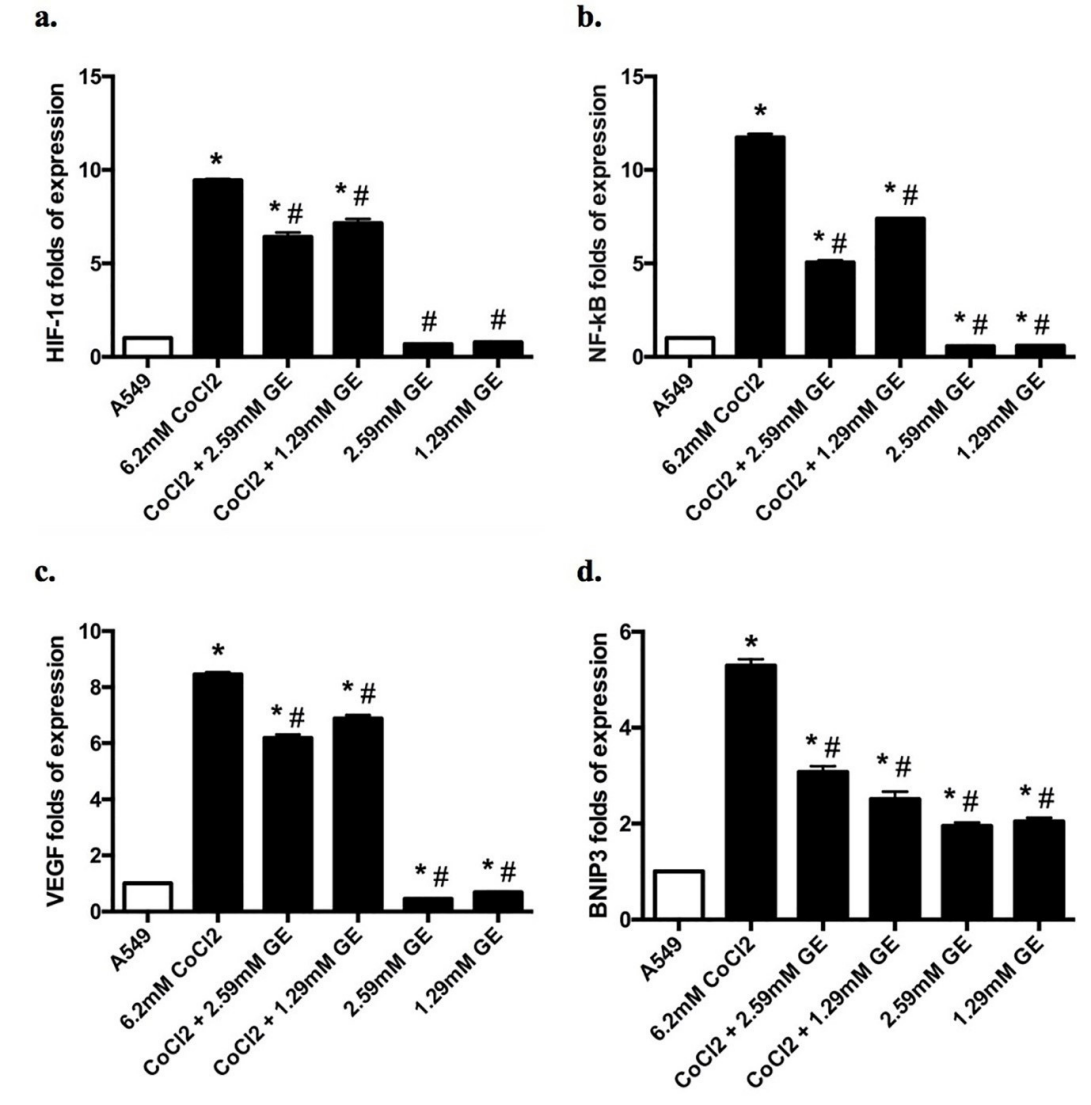
The effect of GE (2.59 and 1.29mM) and CoCl2 (6.2mM) either alone or combined on the expression of HIF-1α, NF-κB, VEGF, BNIP3, and beclin-1 mRNA in A549 cells for 24 h using qRT-PCR. Data expressed as the mean ± SD of relative expression. (*) significantly different from A549 group (*P* < 0.05), (#) significantly different from 6.2mM CoCl2 group (*P* < 0.05).

As shown in Figure 5a-e, CoCl_2_ treated group showed in a significant upregulation in the mRNA expression levels of HIF-1α (9.4 fold), NF-κB (11.74 folds), VEGF (8.46 folds), BNIP3 (5.3 folds), and beclin-1 (2.7 folds) compared to untreated groups, indicating a significant incidence of hypoxia, autophagy as well as angiogenesis. On the other hand, combined therapy of GE (2.59 and 1.29mM) and CoCl_2_ showed a significant downregulation in the mRNA expression levels HIF-1α (6.4 and 7.15 folds respectively), NF-κB (5.1 and 7.4 folds respectively), VEGF (6.1 and 6.9 folds respectively), and BNIP3 (3.1 and 2.5 folds respectively) as well as a modest downregulation in the mRNA of beclin-1 expression (2 and 1.9 folds respectively) compared to CoCl_2_ treated group. It is worthwhile to mention that GE treated groups showed a significant upregulation in the mRNA expression levels of BNIP3 (1.95 and 2.05 folds respectively), and beclin-1 (4.54 and 2.01 folds respectively) compared to that noticed in untreated groups.

## Discussion

Exploration of antihypoxic agents from plant origin has drawn great attention these years in cancer research. From MTT results, GE induced cytotoxicity and loss of A549 cell viability at a concentration of 2.59mM (IC_50_ value). Yet, Galle and colleagues reported that the IC_50_value of GE was 797.2 μM.^29^ Also, CoCl_2_ is a hypoxic imitator agent that induces structural modification in the heme protein O_2_ sensor that results in the generation of ROS, which provokes oxidative stress resulting in hypoxia-induced cytotoxicity.^39^ In this study, the IC_50_ value of CoCl_2_ was 6.2mM. But Mahey and colleagues reported that 29.81 mg/L (231mM) is the IC_50_ value of CoCl_2_.^40^

ROS formation in the mitochondria during hypoxia aggravates the oxidative stress, induces excessive autophagy, and disrupts the oxidant/antioxidant balance within the cells.^41,42^ This study showed that the IC_50_ value of CoCl_2_ (6.2mM) disrupted the oxidant/antioxidant balance in A549 cells, which results in the incidence of lipid peroxidation with a subsequent reduction in the TAC level. This finding further corroborates earlier observations of Tripathi et al^32^ as well as Mohamed et al.^43^ GE (2.59 and 1.59mM) possesses antioxidant capacity when combined with CoCl_2_ by reducing the level of MDA parallels with the elevation in the TAC level. This finding is similar to that of Khan and colleagues.^21^ The antioxidant activity of GE results from increasing endogenous antioxidant defense system through the elevation in glutathione peroxidase and superoxide dismutase activity via prompting their transcriptional up-regulation.^22,44^

HIF-1α is released during hypoxia to maintain oxygen homeostasis besides the upregulation of transcriptional factor NF-𝜅B; both are factors of stress response to tumor-associated hypoxia.^45^ Recent studies reported a bi-directional relation between HIF-1 α and NF-κB because HIF regulates the expression of NF-κB mRNA and vice versa.^8,45^ Also, HIF and NF-κB activate the expression of several genes controlling angiogenesis (VEGF), cytokines (tumor necrosis factor alpha), chemokines (interleukin-8), and cell death related proteins (Noxa and BNIP3).^45^ Under stress condition such as hypoxia, NF-κB nexuses autophagy and inflammation through activation of mTOR, which in turn stimulates HIF-1 α and NF-κB transcriptional activation results in a sequence of hypoxic and inflammatory cascades that ends in autophagy and inflammation.^46^ Together, these findings further support the results of our study where CoCl_2_ treated cells showed a rapid upregulation in mRNA of HIF-1α, NF-𝜅B, VEGF, BNIP3, and beclin-1 expression. Accordingly, these results indicated the rapid induction of hypoxia with angiogenesis that is concurrent with autophagy, and this agrees with other researchers using a similar approach.^43,47,48^ The most consistent effect of CoCl_2_ to accumulate HIF-1α is through the direct binding of cobalt with oxygen-dependent degradation domain of HIF-α, followed by the suppression of hydroxylated HIF-1α to be produced, as well as its interaction with von Hippel-Lindau protein.^49^ Also, transcriptional activation of HIF-1α induced by CoCl_2_ results in the direct activation of BNIP3 (hypoxia response element), induced programmed cell death as well as regulates autophagy through hindering the binding between Bcl-2 and beclin-1.^50,51^

GE is a natural product that blocks the modification of IκB which results in the inhibition of NF-κB activation as well as its transcriptional activation in TPA treated mouse skin.^21^ Also, GE possesses a strong antiangiogenic, anti-inflammatory, antiproliferative, and apoptosis effect through the downregulation of VEGF, NF-κB, cyclin D1, PCNA, c-fos, p53 and Bcl-2 in buccal pouch carcinogenesis.^52^ Moreover, GE suppresses the AKT signal with the activation of the AMPK pathway, followed by the suppression of mTOR phosphorylation that results in autophagy in PC-3 cells.^53^ Furthermore, GE elevated the BNIP3 and BAX (Bcl-2 family members, apoptotic markers) as well as downregulated the cell cycle regulators (cyclins and CDKs) in PC-3 cells.^53^ Inhibition of HIF-1αcan suppress autophagy by reducing the amount of LC3-II and LC3-I in oral squamous cell carcinoma.^54^ Together, these findings further support the results of this study. Combination therapy of GE (2.59 and 1.59mM) with CoCl_2_ promotes the down-regulation of HIF-1α and NF-κB expression that consequently downregulates the angiogenic expression factor (VEGF); reduction of autophagy was induced through the downregulation of BNIP3 with a modest downregulation in beclin-1 expression. It is important to mention that GE treated groups showed a significant upregulation in BNIP3 and beclin-1 expression indicates that the ability of GE to induce autophagy via the BNIP3/beclin-1 signaling pathway. Our results corroborate the observations of Kim and colleagues who reported that GE affects the expression of BNIP3 in PC-3 cells.^53^ Still, there are no reports on the effect of GE on HIF-1α in hypoxia and how it connected to autophagy through the BNIP3/beclin-1 signaling pathway.

Autophagy and apoptosis share many regulators and interactions like beclin-1/Bcl-2,^13^ and caspase/beclin-1cleavage.^14^ Apoptosis induced by insufficient and excessive autophagy that resulted from the accumulation of autophagic vacuoles.^55^ Also, inhibition of autophagy was accompanied by apoptotic cell death in colorectal cancer cells.^56^ This study showed that CoCl_2_ treated group induced a significant increase in the percentage of apoptotic cells compared to that of necrotic cells as well as upregulation in the expression levels of BNIP3 and beclin-1. Cobalt ion possesses a cytocidal activity and induces hypoxia followed by the upregulation and downregulation of apoptotic and anti-apoptotic proteins subsequently, which stimulates apoptotic cell death.^32^ Also, Chen et al showed that CoCl_2_ upregulates the HIF-1α as well as autophagic proteins (LC3, BNIP3, and beclin-1) in the C2C12-hypoxic model.^51^ These reports and our current results confirmed the link between CoCl_2_, hypoxia, autophagy signaling, and apoptotic cell death.

Moreover, GE in the combination therapy with CoCl_2_ showed a significantly higher percentage of apoptotic cells and a down regulation in the BNIP3/beclin-1 level. Also, GE alone showed a significant percentage of apoptotic cells, an upregulation in the BNIP3/beclin-1 level. Our results corroborate the observations of Kim and colleagues who reported the GE induced autophagy and apoptotic cell death in PC-3 cell.^53^ BNIP3 (Bcl-2 19KD interacting protein) is a mitochondrial outer membrane protein that is activated under hypoxia by the action of HIF-1 to clear the impaired mitochondria; therefore driving autophagy by acting as a mitophagy receptor.^50^ Furthermore, BNIP3 can avert the connection between beclin-1 and Bcl-2, freeing beclin-1 to hasten the autophagic process through autophagosome formation. This in turn induces apoptosis by increasing mitochondrial membrane permeability.^18-20^ Also, beclin-1 overexpression by an anticancer drug resulted in apoptotic cell death in cervical cancer cells.^5^

## Conclusion

In this study, we assessed the antihypoxic role of GE (2.59 and 1.29mM) against CoCl_2_ induced hypoxia in the A549 cell line which relies on restoring pro-oxidant/antioxidant equilibrium. Also, GE modulates the inflammatory and angiogenic markers through downregulation of the expression levels of NF-𝜅B and VEGF that results from the upregulation of HIF-1α during hypoxia. Additionally, GE reduces autophagy in combination therapy by downregulating the expression levels of BNIP3 and beclin-1, associated with an elevation in apoptotic cell death through HIF-1α signaling pathways. Further experiments will be needed to explore the mechanism of GE in controlling hypoxia-associated autophagy at the molecular and cellular levels in different cell types.

## Ethical Issues

Not applicable.

## Conflict of Interest

The authors declare no potential conflicts of interest.
